# Lymphatic Vessel Invasion in Routine Pathology Reports of Papillary Thyroid Cancer

**DOI:** 10.3389/fmed.2022.841550

**Published:** 2022-02-21

**Authors:** Costanza Chiapponi, Hakan Alakus, Matthias Schmidt, Michael Faust, Christiane J. Bruns, Reinhard Büttner, Marie-Lisa Eich, Anne M. Schultheis

**Affiliations:** ^1^Department of General, Visceral, Cancer and Transplant Surgery, University Clinic of Cologne, Cologne, Germany; ^2^Department for Nuclear Medicine, University Clinic of Cologne, Cologne, Germany; ^3^Policlinic for Endocrinology, Diabetes and Prevention Medicine, University Clinic of Cologne, Cologne, Germany; ^4^Institute for Pathology, University Clinic of Cologne, Cologne, Germany

**Keywords:** papillary thyroid cancer, lymph node metastasis, lymph vascular invasion, nodal involvement of papillary thyroid cancer, L0, L1, lymph vessel

## Abstract

**Purpose:**

It is not mandatory to report lymphatic vessel invasion in pathology reports of papillary thyroid cancer (PTC) according to the current Union for International Cancer Control (UICC) TNM (tumor, nodes, and metastases) classification. However, there is some evidence for its correlation with lymph node metastasis (LNM) and prognosis. The aim of this study was to explore the clinical implication of lymphatic vessel invasion documentation of PTC because pathology reports play a pivotal role in postsurgical clinical decision-making in endocrine tumor boards.

**Methods:**

Patients undergoing postoperative radioiodine treatment for PTC at the University Hospital of Cologne, Germany between December 2015 and March 2020 were identified. Pathology reports were screened for documentation of lymphatic vessel invasion. Demographics and clinicopathologic data of patients documented, including lymphatic vessel invasion and lymph nodal involvement were analyzed.

**Results:**

A total of 578 patients were identified and included. Lymphatic vessel invasion was reported in pathology reports of 366 (63.3%) and omitted in 112 (36.7%) patients. Positive lymphatic vessel invasion (L1) was diagnosed in 67 (18.3%) of 366 patients and was documented as absent (L0) in 299 (81.7%) patients. Lymph nodal (N) status was positive (N+) in 126 (45.6%) and negative (N0) in 150 (54.3%) of these patients. In 54 (80.6%) L1 cases N+ status and in 137 (65.6%) L0 cases N0 status was diagnosed. In 13 (19.4%) cases with L1 status, there were no LNMs (L1 N0). In total, 72 (34.4%) patients had LNM despite L0 status (L0 N+). The sensitivity and specificity of LVI reporting for LNM were 0.42 and 0.91, respectively.

**Conclusion:**

In routine pathology reports of PTC used for indication to postoperative radioiodine treatment by a German endocrine tumor board, lymphatic vessel invasion was found to be reported inconsistently and mostly as L0. L1 diagnoses, however, reliably correlated with reported LNM and might, thus, be relevant for clinical decision-making. For this reason, we advocate for standardized pathologic reassessment of lymphatic vessel invasion, in particular for cases where lymph nodes are not included in the pathologic specimen and if L0 is documented.

## Introduction

Papillary thyroid cancer (PTC) has a very good prognosis. For this reason, the extent of surgery and prophylactic lymphadenectomy are controversial issues. The European Society of Endocrine Surgery (ESES) recommends prophylactic lymphadenectomy in patients with tumor stages T3 or T4, in patients older than 45 years or younger than 15 years, of the male gender, with bilateral or multifocal tumors, and known involved lateral lymph nodes (1). The British Thyroid Association (BTA) advocates for it in aggressive histopathological subtypes, in patients older than 44 years, with multifocality and tumors sized >4 cm at their largest diameter (2). Also, according to the American Thyroid Association (ATA) “prophylactic central-compartment neck dissection (ipsilateral or bilateral) should be considered in patients with papillary thyroid carcinoma with clinically uninvolved central neck lymph nodes (cN0) who have advanced primary tumors (T3 or T4) or clinically involved lateral neck nodes (cN1b), or if the information will be used to plan further steps in therapy” (3). The European Society of Medical Oncologists (ESMO) underlines that it facilitates the precise staging of the disease and guides subsequent treatment and follow-up, although there is controversial evidence supporting the improvement of recurrence or mortality rate (4). Moreover, the Japanese Association of Endocrine Surgeons (JAES) recommends it for all patients with PTC, excluding papillary microcarcinoma of the thyroid (5). Personalized approaches are required, taking into account the context of the patient, the tumor biology, the experience of the thyroid surgeon, and the benefit/risk ratio (6). In a recent meta-analysis including 9,369 PTCs, lymph node metastasis (LNM) was identified in 31.7% of patients (7). Significant risk factors were age (<45 years), gender (male), multifocality, tumor size (>1 cm), tumor location (upper third of the thyroid), capsular invasion, and extrathyroidal extension (ETE) (7). However, not negligible rates of LNM have been reported for pT1a tumors (8, 9). The clinical significance of lymph nodal involvement in pT1a and pT1b tumors is controversial. Although a recent study reports rates of 11% for PTCs incidentally diagnosed during autopsy (10), there is also some evidence for an increased risk of recurrence (11) and for compromised overall survival in patients with nodal involvement (12), making this issue very relevant for young patients. For this reason, the diagnosis of LNM leads to radioiodine treatment and suppressive TSH treatment during follow-up, even in pT1a patients in Germany. Since pT1a or even pT1b tumors can be diagnosed incidentally in patients undergoing thyroidectomy for multinodular goiter, many of these patients do not undergo lymphadenectomy.

**Figure 1:**
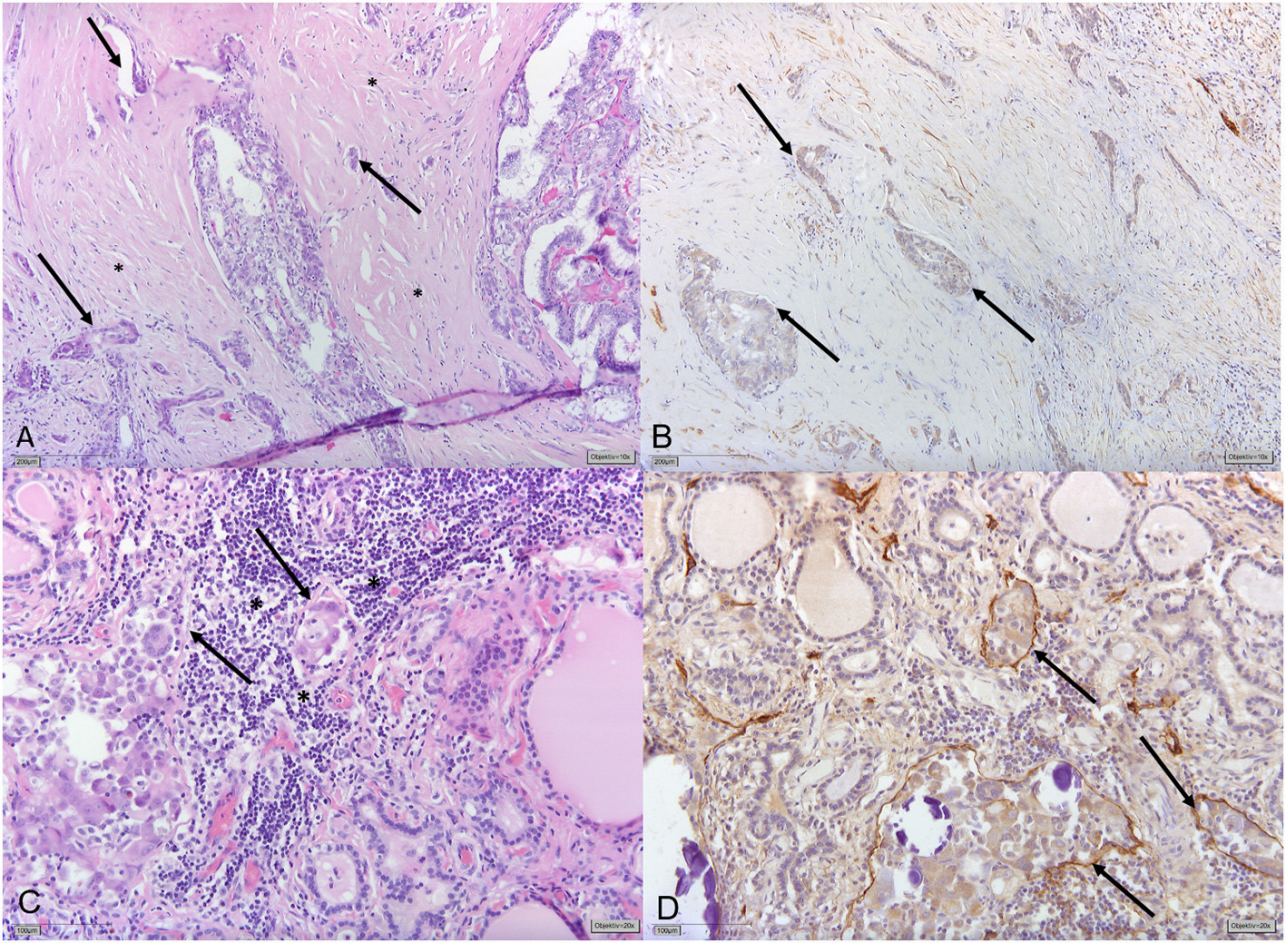
**(A)** Papillary thyroid carcinoma (PTC) displaying significant stromal desmoplasia (*) with small tumor foci surrounded by slit-like spaces (→) indicating vascular invasion. **(B)** Immunohistochemical staining for Podoplanin/D2-40 shows that slit-like spaces lack circumferential staining for D2-40 (→) indicating the presence of retraction artifacts and not lymphatic vessel invasion (LVI). **(C)** Different tumor areas of the same PTC show small tumor nodules with discrete slit-like spaces (→) surrounded by lymphocytes (*). **(D)** Circumferential D2-40-positivity confirming LVI (→).

Lymphatic vessel invasion (LVI) has been shown to correlate with LNM and with patients' prognoses in several tumor entities (13–16). In a recent study, it was found to be the only independent prognostic factor of disease free survival (DFS) in patients with lymph node-negative superficial esophageal squamous cell carcinoma (17). Even its documented absence (L0) might play some predictive role: a multicenter retrospective analysis found that no LNMs were identified in women with low-risk cervical cancer and no LVI. L0 was suggested as a possible argument for omitting prophylactic lymphadenectomy in these patients (18). Although there is some evidence that LVI in PTC correlates with LNMs (19–22) and with outcomes of patients (6, 23, 24), LVI does not play any concrete role in clinical decision-making. Vascular invasion instead is included among those crucial histologic variables for initial risk stratification and clinical management of PTC, alongside ETE, margin status (R), and the number of metastatic lymph nodes (pN-status) (25–27). LVI (L-status; L1 = invasion and L0 = no invasion) is not regularly included in pathology reports in PTC in Europe, as it is not mandatory to report LVI according to the current TNM classification (28). One possible explanation is that LVI in PTC is often not easily identified in thyroid parenchyma (Figure 1) (29).

Lymphatic vessel invasion is defined as “*tumor deposits within lymphatic spaces*” that may manifest “*as psammoma bodies alone within these spaces*” (27, 30, 31). However, often the invaded lymph vascular channels are overgrown by the tumor, as evidenced by the absence of lymphatic channels in the central part of most PTCs with lymph node metastases (29). The thin walls of the lymphatic vascular channels and the invasive nature of the tumors are factors, which can make LVI detection challenging (Figure 1). In addition, LVI assessment is not sufficiently standardized in our experience: for the most part, only representative tumor sections are embedded and H&E staining of representative sections are deemed sufficient for reporting the lack of LVI (L0). However, LVI is not only limited to the tumor itself and tumor interface but could be manifested by the spread of PTC in the ipsilateral and even contralateral lobe seen as psammomas and/or (usually subcapsular) “tumor seeds.” Additional immunohistochemical stains or additional tissue sectioning might help detect LVI. Both, generally, are not routinely performed in the clinical setting (Supplementary Figure 1).

The aim of this study was to critically question the LVI documentation in routine pathology reports of papillary thyroid cancer. Tumors that were deemed worth treating with radioiodine therapy by the multidisciplinary endocrine tumor board of the university were included and the impact of LVI documentation on clinical and pathological parameters was explored. We also investigated if LVI documentation might be helpful for identifying nodal involvement in pT1a tumors.

## Methods

### Patients

Patients who underwent postoperative radioiodine therapy between December 2015 and March 2020 (4 years and 3 months) for PTC as judged by the interdisciplinary tumor board for endocrine tumors at the University Hospital Cologne, Germany were identified and included. In Germany, national guidelines advise for radioiodine therapy in PTC pT1b and higher, and in selected cases of pT1a (e.g., unfavorable histology, lymph node involvement, etc.). Postoperative tumor board recommendations are generally based on pathology reports besides clinical information delivered by the presenting physician.

Surgery was performed as thyroidectomy with or without central lymph node dissection depending on the time of diagnosis (pre- or postoperatively) and the experience of surgeon, as recommended by the German Guidelines (32). In some cases, lateral lymph node dissection was also performed, when the preoperative radiologic diagnosis suggested lymph node involvement, according to the current German Guidelines (32).

### Histopathology

In our pathologic institute, the resected specimens were fixed in 4% phosphate-buffered formalin and embedded in Paraffin. For each cm of the tumor (largest diameter), one tumor section was embedded. A 3-cm large tumor would be analyzed using three tumor sections, ideally comprising one entire diametrical section of the tumor. Three micron thick sections were cut and stained with H&E. Diagnoses were made according to the, at that time, current (2009 and 2017) WHO classification of Tumors of Endocrine Organs. Shortly, H&E-stained sections were routinely screened for signs of LVI. These included the presence of tumor tissue and/or psammoma bodies in lymphatic spaces, including lymphatic spaces within the tumor, but also at the periphery of the tumor, or somewhere else in the resection specimen, as described by Mete et al. (27) and Wittekind (28). Additional immunohistochemical staining was not routinely performed, but only in a subset of cases, which were deemed unclear. Staging, including the assessment of the Nodal Status and LVI, was performed according to the 2018 International Union Against Cancer (UICC) TNM classification system (28). In addition to the number of the resected and metastatic lymph nodes, the number of metastatic lymph nodes with extracapsular extension (ece+) was also determined.

In instances where patients were only referred for radioiodine treatment to our University Hospital, only pathology reports were obtained and included.

Additionally, in order to find out if LVI identification might be helpful for identifying nodal involvement in pT1a tumors, 22 specimens of papillary microcarcinomas with confirmed nodal involvement were retrospectively reassessed with H&E and D2-40 immunohistochemistry (IHC) by two experienced pathologists, according to the criteria defined above (28) (presence of tumor tissue and/or psammoma bodies in lymphatic spaces, including lymphatic spaces within the tumor, but also at the periphery of the tumor, or somewhere else in the resection specimen).

### Data Collection, Analysis, and Ethic

Electronic and paper data of the University Hospital of Cologne were retrospectively collected and analyzed. Data were analyzed using Excel 365 and IBM SPSS Statistics for Windows, Version 25.0, Armonk, NY, USA. This study was approved by the ethics committee of the University Hospital Cologne (Approval ID 20-1724).

## Results

### Characteristics of Patients

Between December 2015 and March 2020, 740 patients received radioiodine treatment at our institution. Forty (5.4%) cases were excluded due to incomplete data. Five hundred and seventy-eight (82.6%) reports described papillary thyroid carcinomas (PTC) and were included in the present analysis. They corresponded to 175 (30.2%) males and 403 (69.8%) females. The median age of male patients was 50 years (range 17–82 years) and that of female patients 47 years (range 11–85 years) (*p* < 0.05). Advanced tumor stages (pT3/T4) were diagnosed more frequently (*p* < 0.05) in men (42 of 175 cases, 42%) than in women (63 of 403, 15.6%).

### Lymphatic Vessel Invasion Report

In 366 (63.3%) of 578 pathology reports, the LVI status was described. The frequency of LVI reporting was not associated with gender of patients (*p* = 0.54), age (*p* = 0.37), tumor size (*p* = 0.11), PTC variant (*p* = 0.57), or pT status (*p* = 0.66). LVI status was more often reported in patients without N-reporting (Nx, *p* < 0.05) (Table 1).

**Table 1:** Lymphatic vessel invasion (LVI) report and clinicopathologic variables (*p-values are calculated for LVI reported vs. non-reported).

		**LVI report**		
	**∑** ***n*** **= 578**	**LVI reported** ***n*** **= 366** **(81.7%)**	**LVI non-reported** ***n*** **= 212** **(18.3%)**	***p*** *
**Age**				
Median (range)	47.5 (11–85)	48 (11–85)	49 (18–82)	n.s.
**Gender**				
Male	175 (30.3%)	113 (64.6%)	62 (35.4%)	n.s.
Female	403 (69.7%)	253 (62.7%)	150 (37.3%)	
**Tumor size**				
Median (mm)	18.5 (11–79)	20 (11–75)	18 (11–79)	n.s.
**PTC variant**				
Conventional	495 (85.6%)	315 (63.6%)	180 (36.3%)	
Follicular	7 (12.6%)	45 (61.6%)	28 (38.4%)	
Tall cell	6 (1.0%)	3 (50.0%)	3 (50.0%)	n.s.
Columnar	3 (0.5%)	3 (100.0%)	0	
Cribriform morular	1 (0.2%)	0	1 (100.0%)	
**pT status**				
1a	135 (23.3%)	79 (58.6%)	56 (41.4%)	
1b	205 (35.4%)	127 (62.0%)	78 (38.0%)	
2	133 (23%)	98 (73.7%)	35 (26.3%)	n.s.
3	88 (15.2%)	53 (60.2%)	35 (39.8%)	
4	17 (2.9%)	9 (53.0%)	8 (47.0%)	
**pN category**				
N0	256 (44.3%)	150 (58.6%)	106 (41.4%)	
N+	198 (34.2%)	126 (63.6%)	72 (36.3%)	<0.05
No lymph nodes resected	124 (21.4%)	90 (72.6%)	34 (27.4%)	

*Tumor size was calculated for the non-pT1a tumors. Next to the median values, the ranges are reported. n.s., non-significant*.

### Lymphatic Vessel Invasion Status

Lymphatic vessel invasion absence (L0) was documented in 299 (81.7%) and positive lymph vascular invasion (L1) in 67 (18.3%) of 366 pathology reports. Lymph vascular invasion (L1) was more frequently detected in male patients (*p* = 0.01), greater tumor size (*p* < 0.05), higher pT- (*p* < 0.01), and pN-status (*p* < 0.01). Whereas, lymph vascular invasion (L1) was seen in only 5% of patients with a pT1a status, the frequency of L1 increased to 23.5% in pT2, 32% in pT3 and 66.7% in pT4 (*p* < 0.01). Similarly, lymph vascular invasion (L1) was reported in only 8.7% patients with pN0 and in 42.8% patients with pN+ (*p* < 0.01). In Nx patients L1 was reported in only 4.4% of cases (*p* < 0.01) (Table 2).

**Table 2:** LVI presence (L1) and absence (L0) and clinicopathologic variables (^*^p-values are calculated for L0 vs. L1).

		**LVI status**		
	**∑** ***n*** **= 366**	**L0** ***n*** **= 299** **(75.7%)**	**L1** ***n*** **= 67** **(24.3%)**	***p*** *
**Age**				
Median (range)	48 (11–85)	48 (17–85)	46 (11–76)	n.s.
**Gender**				
Male	113 (30.9%)	84 (74.3%)	29 (25.7%)	<0.01
Female	253 (69.1%)	215 (84.9%)	38 (15.0%)	
**Tumor size**				
Median (mm)	20 (11–75)	18 (11–64)	25.5 (11–75)	<0.05
**PTC variant**				
Conventional	315 (81.4%)	255 (80.9%)	60 (19%)	
Follicular	45 (12.2%)	41 (91.1%)	4 (8.9%)	
Tall cell	3 (0.8%)	2 (66.7%)	1 (33.3%)	
Columnar	3 (0.8%)	1 (33.3%)	2 (66.7%)	n.s.
Cribriform morular	0 (0.0%)	0	0	
**pT status**				
1a	79 (21.5%)	75 (94.9%)	4 (5.0%)	
1b	127 (34.6%)	110 (86.6%)	17 (13.4%)	
2	98 (26.8%)	75 (76.5%)	23 (23.5%)	<0.01
3	53 (14.5%)	36 (68.0%)	17 (32.0%)	
4	9 (2.5%)	3 (33.3%)	6 (66.7%)	
**pN category**				
N0	150 (41.0%)	137 (91.3%)	13 (8.7%)	
N+	126 (34.4%)	72 (57.2%)	54 (42.8%)	<0.01
No lymph nodes resected	90 (24.6%)	86 (95.6%)	4 (4.4%)	

*Tumor size was calculated for the non-pT1a tumors. Next to the median values, the ranges are reported. n.s., non-significant*.

### Lymphatic Vessel Invasion and Lymph Node Metastasis (LNM)

In 454 (78.5%) of 578 PTC pathology reports the N-status was reported either as negative 256 (N0, 56.4%) or positive 198 (N+, 43.6%). In 276 (60.8%) reports, a LVI and a pN-status were also documented (Table 2).

Patients with nodal involvement showed significantly more often an L1 diagnosis (80.6 vs. 19.4%, *p* < 0.01) and an advanced pT status (pT4 88.9 vs. 11.1%, *p* < 0.05) in their pathology report (Supplementary Table 1). They were significantly more frequently male (63.8 vs. 36.2%; *p* < 0.01), younger (median age 41 years, range 11–84 vs. 51, range 18–85; *p* < 0.01) and had a higher number of harvested lymph nodes (21.3 ± 15.8 vs. 14.5 ± 13.9, *p* < 0.01) (Supplementary Table 1). The independent variables LVI, pT status, gender, age, and the number of harvested lymph nodes, which were significant in univariate analysis, were then entered into the regression model for multivariate logistic regression analysis. The results showed that LVI, male gender, younger age, a larger number of harvested lymph nodes were significant risk factors for LNM (Supplementary Table 2).

Positive LVI (L1) was described in 67 (24.3%) patients. Among them, 54 (80.6%) also had positive pN-status (L1pN+) and 13 (19.4%) had a pN0-status (L1pN0).

L0 was documented in 209 (57.1%) patients. Among them, 137 (65.6%) had a pN0-status (L0pN0), whereas in 72 (34.4%) patients a pN+ status was documented (L0pN+). Most L0pN+ cases were found in pT1b (39.2%), followed by pT2 (24.3%) and pT1a (17.5%).

Additionally, 22 pT1a tumors with nodal involvement were reassessed using H&E and D2-40 IHC (Supplementary Figure 1). They had been diagnosed as L0 in 13 (61.9%) cases and as L1 in 2 (9%) cases. In 7 (31.8%) cases, L status was not documented. Retrospective reassessment using both H&E and D2-40 IHC performed by two independent, experienced pathologists delivered no tumor tissue and/or psammoma bodies in lymphatic spaces, including lymphatic spaces within the tumor, but also at the periphery of the tumor, or somewhere else in the resection specimen, and L0 was reassessed according to this definition in all cases (Supplementary Table 2).

### Predictive Value of LVI Report for Lymph Node Metastasis

The positive assessment of LVI (L1) correlated in 80.6% of cases with lymph node metastases. The documented absence of LVI (L0) was correlated in 65.5% of cases with tumor-free lymph nodes. The sensitivity and specificity of the LVI report for lymph nodal involvement in this study were 0.41 and 0.92, respectively. The positive and the negative predictive value were 0.80 and 0.65, respectively.

As described above, reported LVI was a significant independent risk factor for LNM in the multivariate analysis (*p* = 0.001, Supplementary Table 2).

## Discussion

In several tumor entities, LVI has been shown to correlate with LNMs and with prognoses of patients (13–18). Similar data are available for PTC (6, 19–24). However, LVI does not play a role in clinical decision-making according to all major guidelines and is not mandatory in pathology reports of PTC according to the current TNM classification (28). In Australia, it has been shown to be routinely reported and is only omitted in 15.8% of cases (33). In this study, the data of a German academic endocrine center were explored in order to identify the extent of LVI documentation in routine pathology reports of papillary thyroid cancer and estimate its role as a possible predictor for LNM, especially in cases that generally do not require lymphadenectomy like microcarcinomas <1 cm.

Lymphatic vessel invasion was reported in 63.3% of pathology reports of patients with PTCs, who underwent postoperative radioiodine treatment at our institute. The presence of LVI increased expectedly alongside with higher stage, from 5% in pT1a, 13.4% in pT1b, 23.5% in pT2, 32% in pT3 to 66.7% in pT4 (Table 2). Similarly, the rate of LNM rose from 42.5% in pT1a, 40.8% in pT1b, 44.2% in pT2, 53.5% in pT3, to 88.9% in pT4 (Supplementary Table 1).

Our data confirm that L1 documented in pathology report quite reliably correlated with a diagnosis of lymph node metastases (pN+). A total of 54 (80.6%) patients with reported positive LVI also had a diagnosis of LNM. On the other hand, 13 (19.4%) patients with positive LVI (L1) had a pN0-status in the initial histopathologic report. These L1pN0 cases might be due to the fact that the surgeon performing a prophylactic lymphadenectomy might have missed deeper or lateral lymph nodes, in order to avoid complications in a procedure performed for a cancer, which generally has an excellent prognosis. Eleven (84.6%) of these 13 patients achieved biochemical and radiological cure in a mean follow-up of 27.6 ± 16 months after radioiodine treatment. One 59-year-old woman with a pT1b pN0 (0/1), L1 tumor, and a 31-year-old woman with a pT2 pN0 (0/1), L1 tumor instead have not been cured by initial surgery and radioiodine therapy (RAIT). In the first case, there are elevated and slowly increasing thyroglobulin levels with currently no evidence of structural disease in repeated iodine and PET-CT scan. The second patient underwent repeated cervical surgery delivering eight PTC metastases in 40 resected lymph nodes.

In 72 (34.4%) cases, LNM was reported despite L0 status indicating a concrete risk of lymph node involvement even in L0 reporting. Thirteen of these patients had a pT1a tumor. Pathologic reassessment of 22 specimens including 13 L0, 2 L1, and seven tumors with no LVI documentation was performed by two independent, experienced pathologists. No cases showed LVI diagnosis according to the current definition [presence of tumor tissue and/or psammoma bodies in lymphatic spaces, including lymphatic spaces within the tumor, but also at the periphery of the tumor, or somewhere else in the resection specimen (29)], despite nodal involvement. Two cases, which had originally been reported as L1 (Supplementary Table 3), could not be confirmed as L1. This underlines the difficulty of diagnosing lymph vessel invasion especially in smaller tumors, even with the additional use of IHC.

In the interpretation of the data presented in this study, the reliability of pathology reports needs to be discussed. The thin walls of the lymphatic vascular channels and the invasive nature of the tumors are likely elements masking the evidence of lymphatic invasion (30). Xu and Ghossein recently pointed out that features separating lymphatic from vascular invasion based on histology alone can be difficult (34). Lymphatic invasion is generally assessed by H&E staining method only. One major challenge of this method is to distinguish lymphatic invasion from the retraction artifacts caused by tissue handling and fixation (29) (Figure 1). The use of IHC staining methods can support the diagnosis: a meta-analysis on lymphatic invasion in breast cancer showed detection rates ranging from 10 to 49% for H&E and a narrower range from 21 to 42% for IHC (35). In the daily practice, moreover, only representative parts of the tumor are sectioned and examined, and IHC is not used routinely, indicating a possibility of underdiagnosis of lymphatic invasion (Supplementary Figure 1). Since the CAP 2021 considers LVI as a core reporting element (27), the clear criteria for diagnosis, including direct and indirect LVI signs, should be more broadly implemented in routine pathologic protocols for PTC.

Another issue to consider is the variability in the number of harvested lymph nodes in lymph node dissections. Some pathology reports included only one lymph node and cases were signed out as “pN0” based on this singular, possibly unintended resected node. In several cases lymphadenectomy had not been performed, according to the German guidelines stating that the benefit of prophylactic lymphadenectomy is unclear and should be performed only by experienced thyroid surgeons, weighing the risk of morbidity and the possible benefit for the patient. If lymphadenectomy is performed, there are three main aspects influencing lymphadenectomy results. There are individual patient-associated anatomic aspects, like the normal number of lymph nodes in the central neck compartment varying between 2 and 44 lymph nodes (36). There are also technical aspects, which are relevant to the surgeons and some which are relevant to the pathologist. The surgeon must make sure that the extent of resection does not unnecessarily increase morbidity and the pathologist must be aware that smaller lymph nodes may be hard to detect within large dissection specimens and ideally embed the complete specimen (36). A retrospective Surveillance, Epidemiology, and End Results (SEER) analysis, including the results of 5,107 central lymphadenectomies for PTC, reported an average total number of lymph nodes removed of 4 (37). The significantly higher number reported in this study is possibly due to the fact that most patients receiving lymphadenectomy had been operated in specialized clinics. Additionally, according to the German guidelines, lateral lymphadenectomy is also warranted if the lymph nodes appear sonographically enlarged or suspicious. While evidence of LNMs can help the nuclear medicine specialist in correct staging and for adjusting the radioiodine activity to administer (38), a diagnosis of four tumor-free lymph nodes, instead, can hardly rule out lymph node involvement.

Several studies agree that LVI has a positive correlation with LNMs (19–22), and a negative impact on outcomes of patient (6, 23, 24), although single studies report no significant association with response to therapy (39). In this study, the positive correlation with LNMs can be confirmed. An additional finding is the remarkably high percentage of LNM (34.4%) in L0 tumors, which is also confirmed by the data of Pontius et al. (32.5%) (23). Given this high rate of LNMs, despite the lack of LVI and given the limits of the currently widely used methods (H&E staining of representative parts of the tumors only), the L0 report appears of poor clinical significance.

Some shortcomings of this study must be mentioned. First, the reference standard consisted of pathology reports, thus conclusions might be affected by the individual bias of pathologists, who initially signed out the specimens. So far, we did not routinely reassess specimens of patients referred for postoperative treatment at our institution. Based on the data presented, we plan to reassess lymph vessel invasion in the future, in order to investigate if this information might impact clinical decision-making. Second, long-term follow-up data for some of these patients are not included, hence we did not analyze systematically which of these patients developed metastases in the course of their disease or even radioiodine refractory disease. Finally, the patients included in this study were treated or referred to a university center (40). It also needs to be mentioned that active surveillance for low-risk PTC has shown to be safe and feasible in certain populations. However, in Europe and especially in the time lapse of this study, it is currently limited to clinical trials. Moreover, to the best of our knowledge, there are no data on active surveillance of nodal involvement.

## Conclusion

We conclude that the presence of LVI (L1) documented in routine pathology reports delivers important information, as it likely correlates with LNM. The reported absence of LVI (L0) however, with the widely used H&E staining of a representative section of the tumor, does not reliably correlate with the absence of LNM and might suggest wrong assumptions. In the absence of lymph nodes in the surgical specimen, standardized pathologic reassessment of L0 diagnosis included in routine pathology reports might be helpful for better assessment of the risk of nodal involvement.

## Data Availability Statement

The raw data supporting the conclusions of this article will be made available by the authors, without undue reservation.

## Ethics Statement

The studies involving human participants were reviewed and approved by the Ethic Board of the University Hospital of Cologne. Written informed consent for participation was not required for this study in accordance with the national legislation and the institutional requirements.

## Author Contributions

CC and AS designed the study, together with CJB and RB. CC, M-LE, and AS collected the data. CC, HA, M-LE, MS, MF, and AS contributed to the interpretation of the results. MS, HA, CJB, RB, and MF contributed critical feedback and helped shape the research, analysis, and manuscript. CC and AS wrote the first draft of the manuscript. All authors critically revised the manuscript, approved the final version of the manuscript, decided to submit this study, and agreed to be accountable for all aspects of the work as recommended by the International Committee of Medical Journal Editors (ICMJE) authorship criteria.

## Conflict of Interest

The authors declare that the research was conducted in the absence of any commercial or financial relationships that could be construed as a potential conflict of interest.

## Publisher's Note

All claims expressed in this article are solely those of the authors and do not necessarily represent those of their affiliated organizations, or those of the publisher, the editors and the reviewers. Any product that may be evaluated in this article, or claim that may be made by its manufacturer, is not guaranteed or endorsed by the publisher.

## Supplementary Material

Supplementary Figure 1 Representative cases of papillary thyroid carcinoma depicting the need for standardized LVI evaluation. **(A)** Papillary thyroid carcinoma (PTC; pT1b) without evidence of lymphovascular invasion (LVI) on H&E-stained section of the primary tumor. However, psammoma bodies can be appreciated (*) sometimes surrounded by slit-like spaces. No additional staining was performed. The case was signed out as L1 as a lymph node metastasis was detected [pN1 (4/5)]. **(B)** Hereinafter performed Podoplanin (D2-40) immunohistochemistry (IHC) showed very few lymphovascular channels (→) at the periphery of the tumor; no evidence for LVI. **(C)** PTC (pT1a) with small tumor nodules at the periphery, some of which are surrounded by slit-like-spaces potentially suggesting LVI on HE stained sections. The case was signed out as L0 without further analysis and showed lymph node metastases [pN1 (2/33)]. **(D)** Subsequently performed D2-40 staining depicts vascular channels at the periphery of the tumor without tumor cell invasion supporting the absence of LVI. **(E)** PTC (pT1b) with small foci of tumor nodules surrounded by slit-like spaces at the periphery (*) suggesting LVI. The case was signed out as L1. No lymph node metastases were detected [pN0 (0/6)]. **(F)** LVI could not be confirmed by D2-40 staining.

Supplementary Table 1 Patients with L- and N status. Lymph nodal involvement was significantly more frequent in younger patients, in men and in patients with a higher number of harvested lymph nodes, higher pT status, and lymphatic vessel invasion (LVI) documented in the pathology report. Tumor size was not significant (*p* = 0.47).

Supplementary Table 2 Results of a multivariate analysis including the independent variables gender, age, pT status, LVI and number of harvested lymph nodes, which had been found significant in Supplementary Table 1. The results showed that LVI, gender, age, and a number of harvested lymph nodes were significant risk factors for lymph node metastasis. B, β coefficient; SIG, statistical significance, *p*-value.

Supplementary Table 3 Pathology reassessment of 22 cases of pT1a pN1 papillary thyroid carcinoma (PTCs), including 13 L0, 2 L1, and 7 cases in which LVI had not been documented. LVI could not be diagnosed in any case, also in the two cases in which it had been reported.
