# Supplementary figures and images for: Lymphatic Vessel Invasion in Routine Pathology Reports of Papillary Thyroid Cancer
Source: Front Med (Lausanne). 2022 Feb 21;9:841550. doi: 10.3389/fmed.2022.841550 (PMC8899077; doi:10.3389/fmed.2022.841550)

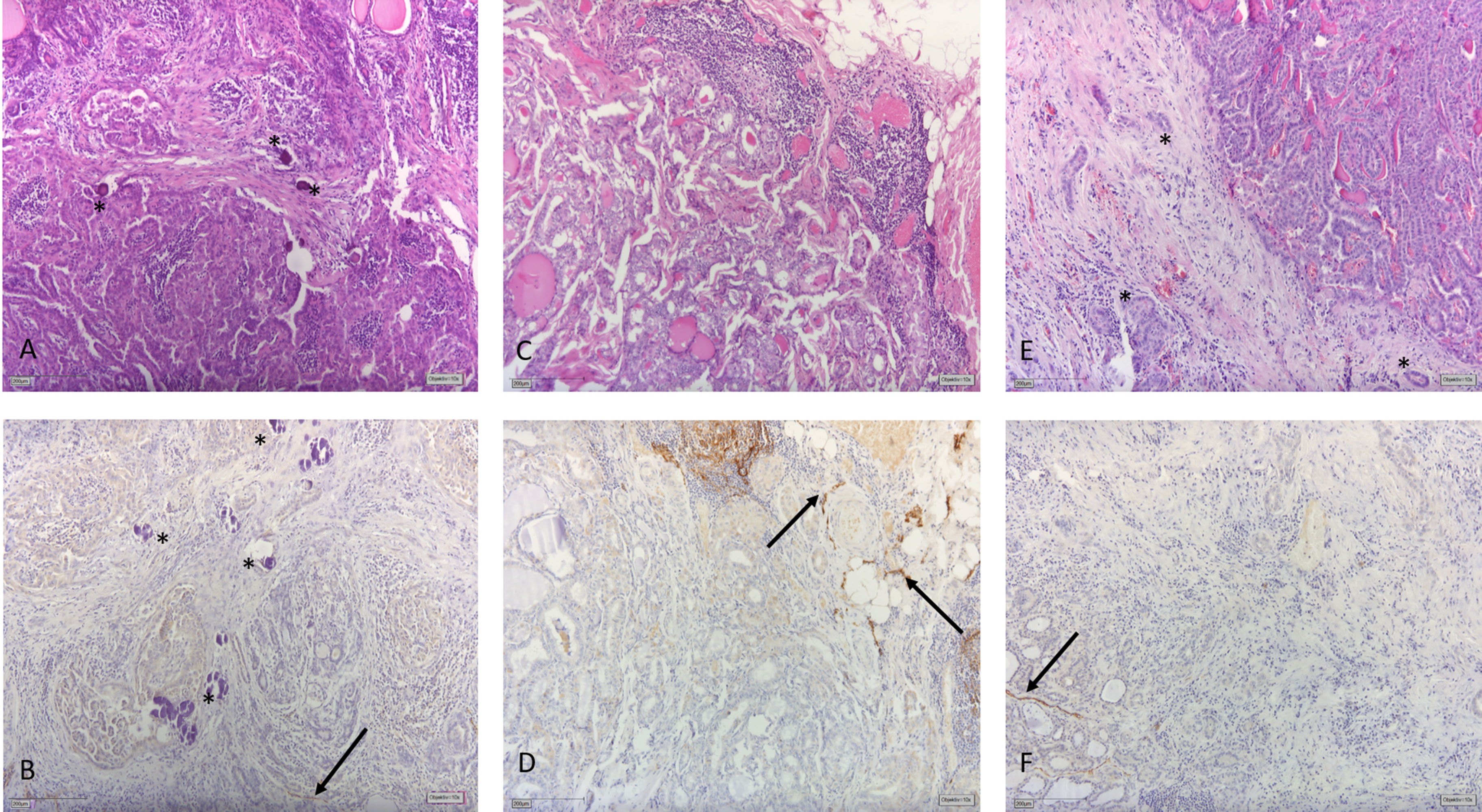

Supplement: Supplementary Figure 1 — Representative cases of papillary thyroid carcinoma depicting the need for standardized LVI evaluation. (A) Papillary thyroid carcinoma (PTC; pT1b) without evidence of lymphovascular invasion (LVI) on H&E-stained section of the primary tumor. However, psammoma bodies can be appreciated (*) sometimes surrounded by slit-like spaces. No additional staining was performed. The case was signed out as L1 as a lymph node metastasis was detected [pN1 (4/5)]. (B) Hereinafter performed Podoplanin (D2-40) immunohistochemistry (IHC) showed very few lymphovascular channels (→) at the periphery of the tumor; no evidence for LVI. (C) PTC (pT1a) with small tumor nodules at the periphery, some of which are surrounded by slit-like-spaces potentially suggesting LVI on HE stained sections. The case was signed out as L0 without further analysis and showed lymph node metastases [pN1 (2/33)]. (D) Subsequently performed D2-40 staining depicts vascular channels at the periphery of the tumor without tumor cell invasion supporting the absence of LVI. (E) PTC (pT1b) with small foci of tumor nodules surrounded by slit-like spaces at the periphery (*) suggesting LVI. The case was signed out as L1. No lymph node metastases were detected [pN0 (0/6)]. (F) LVI could not be confirmed by D2-40 staining. [file Image_1.PNG]
