# Supplementary material for: Lymphatic Vessel Invasion in Routine Pathology Reports of Papillary Thyroid Cancer
Source: Front Med (Lausanne). 2022 Feb 21;9:841550. doi: 10.3389/fmed.2022.841550 (PMC8899077; doi:10.3389/fmed.2022.841550)
Supplement: Supplementary Table 1 — Patients with L- and N status. Lymph nodal involvement was significantly more frequent in younger patients, in men and in patients with a higher number of harvested lymph nodes, higher pT status, and lymphatic vessel invasion (LVI) documented in the pathology report. Tumor size was not significant (p = 0.47). [file Table_1.docx]

**Supplementary Table 1**

|  | | **N status in L1 and L0 PTC** | |  |
| --- | --- | --- | --- | --- |
|  | **∑**  n=276 (%) | **N0**  n =150 (54.3%) | **N+**  n =126 (45.6%) | p* |
| **Age**  Median (range) | 46 (11-85) | 51 (18-85) | 41 (11-84) | <0.01 |
| **Gender**  Male  Female | 83 (30%)  193 (70%) | 30 (36.2%)  120 (62.2%) | 53 (63.8%)  73 (37.8%) | <0.01 |
| **Number of harvested LN**  mean ± standard deviation | 18±13.9 | 14.5±11.3 | 21.3±15.8 | <0.01 |
| **pT status**  1a/b  2  3  4 | 138 (50%)  86 (31.1%)  43 (15.6%)  9 (3.3%) | 81 (58.7%)  48 (55.8%)  20 (46.5%)  1 (11.1%) | 57 (41.3%)  38 (44.2%)  23 (53.5%)  8 (88.9%) | <0.05 |
| **LVI**  L0  L1 | 209 (75.7%)  67 (24.3%) | 137 (65.6%)  13 (19.4%) | 72 (34.4%)  54 (80.6%) | <0.01 |
