# Supplementary material for: Lymphatic Vessel Invasion in Routine Pathology Reports of Papillary Thyroid Cancer
Source: Front Med (Lausanne). 2022 Feb 21;9:841550. doi: 10.3389/fmed.2022.841550 (PMC8899077; doi:10.3389/fmed.2022.841550)
Supplement: Supplementary Table 2 — Results of a multivariate analysis including the independent variables gender, age, pT status, LVI and number of harvested lymph nodes, which had been found significant in Supplementary Table 1. The results showed that LVI, gender, age, and a number of harvested lymph nodes were significant risk factors for lymph node metastasis. B, β coefficient; SIG, statistical significance, p-value. [file Table_2.docx]

**Supplementary Table 2**

|  | **B** | **SE** | **SIG** | **95% CI** |
| --- | --- | --- | --- | --- |
| **LVI** | 0.34 | 0.10 | 0.001 | 0.13 - 0.53 |
| **Gender** | 0.24 | 0.08 | 0.004 | 0.07 - 0.40 |
| **Age** | -0.007 | 0.002 | 0.003 | -0.01 - 0.002 |
| **pT status**  1a/b  2  3  4 | -0.22  -0.33  -0.28  -0.13 | 0.47  0.45  0.47  0.64 | 0.64  0.46  0.54  0.84 | -1.14 - 0.70  -1.22 - 0.55  -1.20 - 0.63  -1.14 - 1.39 |
| **Number of harvested lymph nodes** | 0.008 | 0.002 | 0.0009 | 0.003 - 0.013 |
