# Supplementary material for: Lymphatic Vessel Invasion in Routine Pathology Reports of Papillary Thyroid Cancer
Source: Front Med (Lausanne). 2022 Feb 21;9:841550. doi: 10.3389/fmed.2022.841550 (PMC8899077; doi:10.3389/fmed.2022.841550)
Supplement: Supplementary Table 3 — Pathology reassessment of 22 cases of pT1a pN1 papillary thyroid carcinoma (PTCs), including 13 L0, 2 L1, and 7 cases in which LVI had not been documented. LVI could not be diagnosed in any case, also in the two cases in which it had been reported. [file Table_3.docx]

**Supplementary Table 3**

| Case-ID | **UICC: TNM-classification** | reassessment result |
| --- | --- | --- |
| PTC1 | pT1a, pN1a (1/3), **L0**, V0, R0. | L0 |
| PTC2 | pT1a pN1b (4/23) **L0**, V0 Pn0, R0 | L0 |
| PTC3 | pT1a, pN1a (4/42, ece+), **L0**, V0, Pn0, R0 | L0 |
| PTC4 | pT1a (6 mm), pN1a (2/33), **L0**, V0, Pn0, R0. | L0 |
| PTC5 | pT1a (m), pN1a (6/11), **L0**, V0, R0 | L0 |
| PTC6 | pT1a (m), pN1a (1/18), **L0**, V0, R0 | L0 |
| PTC7 | pT1a (2), pN1b (7/24, ece+), V0 Pn0, R0 | L0 |
| PTC8 | pT1a (2), pN1b (14/31; ece-) - local R0 | L0 |
| PTC9 | pT1a, pN1a (5/28), R0 | L0 |
| PTC10 | pT1a (m = 2), pN1 (1/28), **L0**, V0, Pn0, local R0 | L0 |
| PTC11 | pT1a (m), pN1b (11/41; ece+), **L0**, V0, Pn0, local R0. | L0 |
| PTC12 | pT1a (m; max: 10 mm) pN1b (5/15) - local R0 | L0 |
| PTC13 | pT1a (m)pN1b (1/19 ece-) | L0 |
| PTC14 | pT1a, pN1a (1/5) - local Rx | L0 |
| PTC15 | pT1a (0,5 cm), pN1a (2/74), **L1**, V0, Pn0, R0. | L0 |
| PTC16 | pT1a, pN1b (1/12), local R0. | L0 |
| PTC17 | pT1a (m; max. 8 mm), pN1a (8/27; ece-), **L0**, V0, R0 | L0 |
| PTC18 | pT1a, pN1a (3/20), **L0**, V0, R0. | L0 |
| PTC19 | pT1a (m) (max. 0,5 cm), pN1b (1/1), **L0**, V0, R0. | L0 |
| PTC20 | pT1a (0,5 cm), pN1 (1/5), **L1**, V0, Pn0, R0. | L0 |
| PTC21 | pT1a, pN1a(1/10), R0, V0, **L0** | L0 |
| PTC22 | pT1a (m),pN1(1/8),**L0**,V0,R0. | L0 |
